# Reflecting health: smart mirrors for personalized medicine

**DOI:** 10.1038/s41746-018-0068-7

**Published:** 2018-11-08

**Authors:** Riccardo Miotto, Matteo Danieletto, Jerome R. Scelza, Brian A. Kidd, Joel T. Dudley

**Affiliations:** 1 Institute for Next Generation Healthcare, Icahn School of Medicine at Mount Sinai, New York, NY USA; 2 Department of Genetics and Genomic Sciences, Icahn School of Medicine at Mount Sinai, New York, NY USA

**Keywords:** Health care, Disease prevention, Medical research

## Abstract

Inexpensive embedded computing and the related Internet of Things technologies enable the recent development of smart products that can respond to human needs and improve everyday tasks in an attempt to make traditional environments more “intelligent”. Several projects have augmented mirrors for a range of smarter applications in automobiles and homes. The opportunity to apply smart mirror technology to healthcare to predict and to monitor aspects of health and disease is a natural but mostly underdeveloped idea. We envision that smart mirrors comprising a combination of intelligent hardware and software could identify subtle, yet clinically relevant changes in physique and appearance. Similarly, a smart mirror could record and evaluate body position and motion to identify posture and movement issues, as well as offer feedback for corrective actions. Successful development and implementation of smart mirrors for healthcare applications will require overcoming new challenges in engineering, machine learning, computer vision, and biomedical research. This paper examines the potential uses of smart mirrors in healthcare and explores how this technology might benefit users in various medical environments. We also provide a brief description of the state-of-the-art, including a functional prototype concept developed by our group, and highlight the directions to make this device more mainstream in health-related applications.

## Introduction

Current technology trends are leading the world towards increasing connectivity and shrinking the boundaries between the digital and physical. Substantial streams of information are now gathered and analyzed in the background and then reported at suitable times. Homes provide an example where technical advances can make traditional appliances and other devices more “intelligent” to improve everyday activities.^1^ These smart products contain a combination of hardware and software that permit connections to other devices inside and outside of the house to perform tasks that would otherwise be done manually or using other systems. For example, smart locks can be programmed to grant a person, e.g., a neighbor or friend, access to a house without giving them the key. Embedded sensors can monitor the door passively for unusual events, e.g., the door remains ajar or an unexpected entry, and send an appropriate alert to the owner or authorities. Smart thermostats can adjust the temperature of the house automatically to a preferred setpoint that maintains a pleasant environment and reduces energy costs.

An emerging intelligent house device with potentially wide-ranging applications for healthcare outside of traditional medical settings is the smart mirror. These mirrors augment the reflecting surface that underlies this 8000-year-old technology^2^ with electronic hardware and computer software to provide passive monitoring, reminders, entertainment, information, and many other possibilities.^3^ While applications of smart mirrors have appeared in the automotive^4,5^ and clothing industries,^6^ real-world examples of this technology in healthcare remains limited. Advances in sensors and computing now offer capabilities for making accurate forecasts about meaningful changes in health, monitoring disease progression, and tracking response to treatments, which represent critical areas for innovation in digital health.^7,8^

This paper provides a brief overview of smart mirrors and reports their current status in healthcare. We highlight a few potential applications and describe the new challenges for engineering, machine learning, computer vision, and biomedical research that must be addressed for this new technology to realize some of the promised benefits in various health-related domains. Finally, we outline some directions we believe are necessary to facilitate the large-scale adoption of this device for medical purposes.

## What is a smart mirror?

**Fig. 1:**
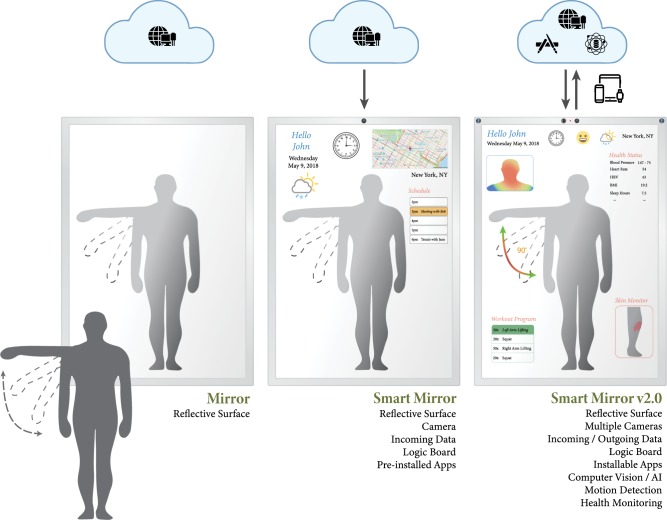
Overview of the potential evolution of mirrors from being a simple reflective surface to a personalized app-based solution deeply interconnected with the cloud as well as all other facets of a user’s digital footprint. The Smart Mirror v2.0 will be able to track activity, passively monitor healthcare status and motion, and couple contextual information, such as weather, schedule and location, with digital biomarkers of health

An ordinary mirror can be transformed into an intelligent artifact, i.e., “smart mirror,” by placing a semi-transparent sheet of glass over a digital screen and connecting this hardware to a computer with incoming data and a camera (Fig. 1). On a basic implementation, the screen can display real-time information about the weather or traffic patterns, alerts from emails, and calendars, as well as data collected via wearables.^9^ This device represents what we call smart mirror version 1.0, yet this simple product offers a substantial upgrade to this common instrument that has otherwise undergone minor innovations in thousands of years. Current consumer versions of this product focus on select markets that augment specific capabilities such as driver assistance^10^ or virtual fitting rooms to try on items of clothing.^11^ Text may appear on a portion of the rear view mirror to aid navigation, and lights may flash on the side view mirrors to alert the driver about objects within their blind spot.^4^ The memory mirror allows people to explore a wardrobe digitally by “wearing” clothes with different colors and patterns projected onto a person’s body reflected in the mirror.^6^

The second generation of smart mirrors fits within a growing set of technologies that integrate monitoring systems with personalized information and computer vision to help individuals achieve their health goals and have a more significant role in healthcare. These devices embed multimodal sensors—multiple cameras, motion detection, lasers, microphones, speakers—as well as software based on artificial intelligence (AI) into the original design and include the capacity to capture and communicate with multiple sources of data, linking them to the broader ecosystem of smart products and the cloud. Such sophisticated yet readily available hardware and software provide the tools to capture physiological measurements non-invasively and to create interactive capabilities based on tracking and recognizing gestures. The smart mirror 2.0 represents a general-purpose platform that multiple stakeholders—engineers, scientists, physicians, hobbyists, the general public—can use to collect large quantities of biomedically relevant data, and to develop a wide range of applications that address many healthcare challenges.

## A smart mirror implementation

**Fig. 2:**
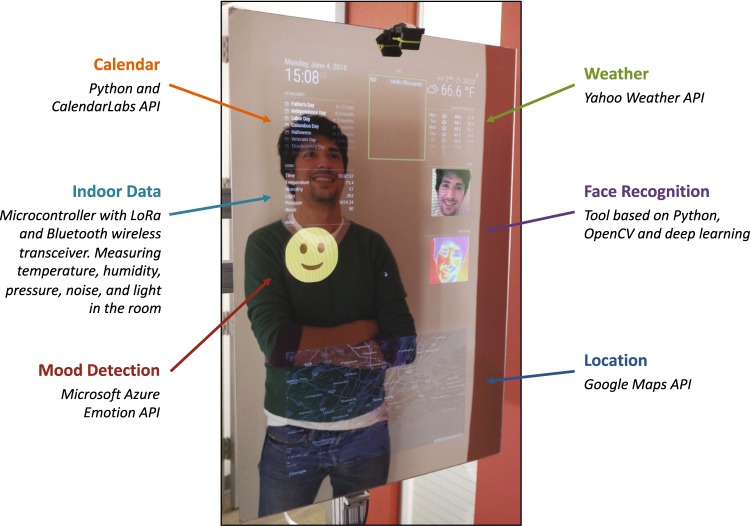
The prototype smart mirror developed in our lab shows a proof-of-concept on leveraging a reflective surface with cameras, Raspberry Pi and online APIs to enhance the user experience by providing information on calendar, weather, indoor data, mood, and location

We set out to build a smart mirror that examined some of the above technical capabilities with potential applications in a clinical or public setting (Fig. 2). We used readily available, off-the-shelf hardware components, and software tools to build our prototype in a few weeks.

Our smart mirror is made from an LCD display covered with a semi-transparent glass. The screen connects to a logic unit based on Raspberry Pi that manages all the functions of the mirror via a GNU/Linux operating system. We followed a plug-and-play paradigm, which prized modularity and usability of third-party open source software. The software infrastructure used a multi-tier architecture that allowed us to examine multiple services that could be relevant for different medical use cases. This scalable architecture enabled us to quickly prototype different features on our smart mirror, such as weather forecasts, environmental data, face recognition, location, mood detection, and breathing rate estimate.

One limitation we encountered in building a smart mirror prototype is we found fewer than anticipated off-the-shelf services for healthcare that we could evaluate. Many features, in fact, still require additional research or technical development. However, we embedded different open source services linked to healthcare, e.g., mood detection as a measure of mental health and facial recognition for personalization. Collectively, we confirmed the feasibility of creating an architecture for smart mirrors that could be tuned by a user based on his/her needs.

## Smart mirrors in biomedical research

The availability of open source software tools and inexpensive, yet sophisticated technologies provides a sandbox for exploring direct applications of smart mirrors in biomedical research as well. Initial studies focused on the feasibility of capturing health metrics and understanding how humans will interact with these new technologies. Preliminary efforts show proof of concept devices that can monitor a range of physiological parameters non-invasively, detect emotional states, and infer an individual’s risk for cardio-metabolic diseases.

*Medical Mirror* encourages people to track their vital signals on a daily basis.^12^ Computer vision captures an optical signal reflected off a person’s face. By analyzing and detecting small deviations in such reflections attributed to pulsating blood flood, this device by Poh and colleagues^13^ provides estimates of an individual's heart rate.

*Fit Mirror* helps people wake up as well as increase their motivation and activity throughout the day.^14^ To shift their mindset, people performed movements in front of this device, which included an interactive display that suggested invigorating physical challenges.

*Wize Mirror* estimated cardio-metabolic risk and anxiety levels from anthropometric measurements of facial features.^15,16^ The concept underlying Wize Mirror is that physical characteristics and facial expressions provide surrogate measures for a person’s health status, all of which can be captured by standing in front of the mirror.

Although all of the devices highlighted above represent research prototypes, their initial demonstrations suggest encouraging results with a clear path toward addressing challenges in healthcare.

## Bridging the gap: developing smart mirrors for healthcare

One takeaway from this sampling of use cases is the small gap between prototype and practice. The consumer-grade hardware and multimodal data analysis tools currently available can achieve results that are useful for both general health enthusiasts and patients.^17,18^ Plus, the trend of publishing specifications for how to build these devices increases the likelihood of finding smart mirrors in the wild that function as personal health assistants. Everyday environments such as houses, gyms, and offices now include prototype devices that provide context-specific information about personal health with nudges toward healthier lifestyles.^19,20^

Recent advances in machine intelligence are creating the opportunities to move from the proof-of-concept stage to reality more quickly. The science that helps machines to understand the visual world, known as computer vision, is rapidly improving from advances in a branch of machine learning that uses a hierarchical computational design inspired by a biologic neuron’s structure, that is deep learning.^21^ Computer vision driven by deep learning algorithms can help inexpensive cameras achieve image-acquisition performances comparable with high-end devices,^22–25^ enabling new opportunities to embed medical-quality image-acquisition in at-home devices, such as mirrors. This shift reflects a significant change because these diagnostic capabilities required expensive machines typically available at hospitals or large medical centers.

Applications of deep learning and computer vision in medicine abound.^26–30^ Two fields where home monitoring with a smart mirror could change the traditional patient-physician interaction include ophthalmology and dermatology. Retinal fundus photographs analyzed by deep learning networks measured potential cardiovascular risk factors, such as gender, age, and systolic blood pressure,^31^ and identified diabetic retinopathy and diabetic macular edema.^32^ One implementation of convolutional neural networks resulted in dermatologist-level classifications of skin cancer from clinical images.^33^ An AI-based dermatologist called SkinVision^34^ showed accurate classifications of skin abnormalities that could indicate malignant growths. Although these examples used a hand-held imaging device, embedding these algorithms into a smart mirror platform would address some of their current limitations such as passive monitoring, removal of hand coordination, and bona fide comparison images captured under more reliable conditions.

Two other digital health domains that can benefit from smart mirrors are anthropometry and functional movement. Defining the relationship between the locations of joints and their position in images is called human pose interpretation. Once again, computer vision and deep learning outperformed the state-of-the-art in detecting, tracking, and deciphering humans and their body pose.^35–37^ These technologies can leverage the smart mirror for a range of healthcare tasks, such as orthopedic diagnostics, physical therapy, personal training, adherence, and health and fitness. For example, the Smart eHealth Mirror design framework captures a person’s posture, conducts an anatomical analysis to determine deviations from optimal alignment, and then provides suggestions for making adjustments to improve individual posture over time.^38^

## Opportunities and benefits

We foresee a near future where smart mirrors represent an integral part of daily life analogous to smartphones. Mirrors offer greater convenience and capabilities for monitoring personal health outside of the traditional medical settings of clinics and hospitals. We believe that five medical applications will benefit the most: passive monitoring, dynamic monitoring, digital biomarker detection, telemedicine, and health and fitness (Table 1). In such categorization, for example, Medical Mirror^12^ and Wize Mirror^15,16^ belong to the “digital biomarker detection” group as they aim to measure heart rate and anxiety level, respectively. Wize Mirror is also a “passive monitoring” device as it provides cardio-metabolic risk. Differently, Fit Mirror^14^ can be considered an example of “health and fitness” application.

**Table 1:** Areas of medicine where the next generation of smart mirrors is likely to create new opportunities to improve the health of patients

Modality	Summary	Application
*Passive monitoring*	The ability to interact with users, without directly engaging them, to monitor physiological changes and health status.	Emotion detection
		Balance measurement
		Skin variation
		Hair loss
		Cardiovascular risk
*Dynamic monitoring*	The ability to receive user operation/interaction as input and provide real-time response related to the input (e.g., correction suggestions, health scores).	Gait analysis
		Cognitive performance
		Grip strength
		Voice tracker
		Physical therapy
*Digital biomarker detection*	The automatic detection of various metrics that are useful for assessing health.	Heart rate
		Heart rate variability
		Blood pressure
		Respiratory rate
		Stress level
		Eye health
*Telemedicine*	A remote interaction between the patient and physicians.	Vitals detection
		EHR integration
		Personalized care
		Health visualization
*Health and fitness*	Fitness and health performance as characterized by general consumer health (not in terms of clinical care).	Weight loss
		Body fat
		Activity tracking
		Motivation
		Metabolic performance
		Personal coaching

For each area, we highlight some specific example applications; we notice that each application can also be combined with other ones to obtain more comprehensive and multimodal systems

An AI-leveraged smart mirror improves both clinical and at-home healthcare. The technology provides easy monitoring and data collection. The data collected from a mirror can link with wearable devices or other smart products to offer daily personal check-ups.^39^ Furthermore, this data can connect to the electronic health records and then be shared with the appropriate healthcare professional, e.g., physician, trainer, physical therapist.^40,41^

Passive monitoring capabilities on the smart mirror can determine baseline conditions and detect deviations that indicate potential changes in health. The device collects relevant measurements while a person uses the mirror as part of their typical routine rather than having to carry (and to recharge) additional hardware. We believe this principle is one of the critical points for increasing the adoption of smart mirrors to improve personal healthcare: *the technology should fit with and support a person’s activities rather than having the individual change their habits to use the new technology*. In such scenario, all the data are silently collected without any obtrusive interaction and provided to the users (or shared with the personal physicians) only when requested (or in presence of anomalies), and potentially as summaries or longitudinal trends. This helps minimize the risk that individuals are inundated with too much information to process, and might decide to tune out some of the signals.

A common household location for a typical mirror is the bathroom. Swapping in a smart mirror permits the collection of health metrics such as body temperature and heart rate, as well as changes in skin features, e.g., color, texture, moles, rashes, while a person goes about their routine. All of these parameters represent meaningful markers of health and this idea represents a second core principle for smart mirror technologies: *at-home monitoring promises new possibilities for more accurate and useful diagnostic metrics in a convenient setting that captures reality more closely*. Smart mobile devices, such as wearables, smartphones, tablets, and laptops, can be used for health monitoring as well, often with no additional costs.^42^ However, individuals are required to actively do the monitoring by setting up the laptop for motion detection, keeping a device always charged for heart rate measurement, or taking pictures of eyeballs or skin areas for ophthalmology and dermatology analysis. Most importantly, users often need to identify that something is wrong with their own body and act based on it. Cameras embedded in the bathroom mirror or elsewhere in the home could help to more passively predict health issues before they manifest clinically. All portable device ecosystem can serve the users for not-at-home monitoring, with these measures integrating the smart mirror data towards more consistent and reliable observations.

Smart mirrors for personal training can replace the regular mirrors in most gyms.^43^ In this context, members could receive guidance, motivation, and encouragement for particular exercises. The same scenario applies to physical therapy, whereby the patient uses biofeedback in performing the recommended exercises. A smart mirror at-home offers the option of recording movements to share this information with the person’s physical therapist and tracking progress.

## Challenges

Given the current technological, clinical, and ethical landscapes, a variety of limitations and challenges must be overcome to realize a number of healthcare benefits from AI-leveraged smart mirrors on a large-scale.

Current hardware is not advanced enough to effectively realize all the potential monitoring opportunities. On one hand, detecting features for dermatology and motion tracking can be done effectively with simple cameras and advanced computer vision techniques.^33,36^ On the other hand, characterizing eye conditions at the standards of ophthalmology or detecting digital biomarkers, such as systolic blood pressure, via deep learning^31^ still requires better devices. Technology solutions exist for capturing retinal fundus images using mobile phones and a hand-held ophthalmoscopy lens.^44^ The technical challenge is to create a mirror camera that can acquire retinal fundus photos automatically without requiring the aid of an additional lens. Imaging technology in this area is progressing fast and it is likely that in the next few years such cameras may be available.^45^ The ability to process multiple computer vision tasks without substantial latency requires more powerful logical boards than what is broadly accessible. Advances in neural engines optimized to run deep learning inferences, together with continuous improvements on GPUs, are likely to have a big part in realizing the envisioned AI-leveraged smart mirror architectures.

The rapid pace of software development created a variety of tools that must be optimized to ensure that trustworthy measurements and the efficient capture of data translate into actionable insights. Most importantly, the reliability and accuracy of the metrics provided by computer vision and machine learning models must be established with large-scale clinical and field trials. Many of the promising technologies reported in the literature require further evaluation. Images collected outside of the lab, i.e., “in the wild,” are likely to be affected by environmental factors, such as different lighting and/or imperfect user positions. Model tuning and sensitivity analysis remain underexplored areas in the context of real-world data. Inaccurate measures might create dangerous precedents if factored into medical decision making, with false negatives causing missed conditions and false positives leading to overtreatment and/or unnecessary anxiety. Similarly, wrong recommendations during physical therapy or gym exercises may lead to longer recoveries or new injuries. Consequently, it is important to establish the appropriate standards and regulations that components and algorithms of a smart mirror must meet.

A major promise for how smart mirrors stand to positively influence healthcare is through the passive collection of a large amount of longitudinal data. While preliminary findings support the analysis of longitudinal data for establishing proper baselines and the continuous monitoring of several conditions relevant for health, e.g., heart conditions, hypertension, and diabetes,^42^ evidence is lacking on how this type of information can be used to change from reactive to preventative medicine. Several initiatives aim to examine this question and establish necessary evidence for shifting healthcare through passive monitoring. The All of Us research program (formerly known as Precision Medicine Initiative),^46^ the MyHeart Counts project^47^ and the Stanford Azumio Activity Inequality study^48^ are all collecting and analyzing large-scale longitudinal measures (from wearable devices) alongside other types of biomedical data from cohorts of 50,000 to one million people. These studies also include clinical and biomolecular information for corroborating health outcomes from more traditional measures. We believe that these initiatives offer the appropriate design framework for uncovering new ways in which longitudinal data can be used to proactively improve healthcare. The lessons from all these efforts will then help build the best practices for more convenient and passive at-home monitor solutions, such as the smart mirror.

The adoption of AI-leveraged smart mirrors might raise some ethical concerns due to the personal nature of the data collected, as well as the actionable steps from this information. Some individuals might not be interested in knowing everything about their health status, but only on some aspects. For example, a user could only be interested in monitoring cardiovascular activities, and not in knowing the Alzheimer’s risk. Strict policies on which extent passive monitoring is performed might be required. In particular, the ability for the users to personalize their smart mirrors by turning on/off some measurements is likely to be a factor in the success of smart mirrors for the house.

## Connection to a broader ecosystem

The smart mirror consolidates multiple functions into a single device. This versatility grows by connecting the device to a digital distribution platform where users browse for software applications, which download and install directly onto the device to augment and customize their experience with the mirror. Analogous to the situation with mobile devices, we foresee developers creating apps and making them accessible via a digital marketplace such as Google Play or the Apple Store. It is not hard to imagine a community of developers creating specific software applications to address healthcare challenges through digital health tools deployed on a smart mirror platform, e.g., skin change detection or rehabilitation exercises.

## Privacy and data sharing

Although many digital health opportunities exist for smart mirror technologies, privacy and data sharing are serious issues that require more attention. Strict access control with a clear understanding of consent around data recording and sharing is an imperative. The mirror’s capability to collect data passively, which may be its most significant feature, could also be its most substantial problem due to potential privacy violations regarding information about an individual’s health. The use of cameras and microphones to collect data, as well as the transfer of these data to cloud storage systems, must be regulated by strict protocols that are currently missing. Privacy should form the cornerstone of the mirror’s hardware and software design architecture. One approach to mitigate potential problems is through implementing a multi-tier privacy plan that includes different steps to process and encrypt data.

We recommend three steps to ensure privacy and safety of the data. First, app approval occurs when the entire production chain and platform development receive security clearance. Second, pre-process the data collected by the mirror locally, and then upload this information to the cloud through a secure and encrypted protocol. Breaking up the data collection, processing, and exchange into a broader ecosystem still allows for aggregate posterior analysis with other cloud-synced data but reduces the risk of sharing sensitive information without permission. On the contrary, such private data remain stored in an encrypted form in the local memory of the device. Third, equip mirrors with a switch, e.g., hardware or vocal, that allows any person to engage or disconnect embedded sensors while retaining the benefit of the reflecting surface. Although this option interrupts passive data collection, a critical component of trust is the ability to control one's privacy. We believe that an architecture based on secure data distribution and storage as well as encryption should ensure the appropriate level of security of user information. The data in the cloud can then be safely analyzed to learn patterns and commonalities that could improve the machine learning-based functionalities of the mirror as well as of its apps. Secure data collection, storage, and transfer standards will also facilitate the integration of smart mirror data with the electronic health records and the usage of at-home monitoring data in the clinic.

## Conclusions

The smart mirror prototypes and concept designs show promise to improve healthcare. What remains uncertain, however, is when or how the prototypes will permeate the marketplace and become an integral component to monitor an individual’s health and to benefit the healthcare system as a whole. We believe that the current digital revolution that leverages deep learning to drive improvements in software and hardware will take the smart mirror to this envisioned wide adoption in health-related applications in the clinic and at-home in the next few years, while maintaining their cost and security at a wide access level.

One pathway for this wide adoption of smart mirrors could start with use in the clinic, and transition towards home environments, following common trends of other medical technologies, such as glucose meters and blood pressure monitors.^49^ Patients start using the device at their doctor’s office, begin to see the benefits of the technology, and consider the possibility of purchasing one for at-home use to save money and trips to a clinic. As an example, potential early use-case scenarios for smart mirrors in the medical setting that can be easily moved at-home are physical rehabilitation monitoring and vital signs measurements.

Addressing these challenges and refining regulations for correct and meaningful uses of the technology will expand the markets and healthcare applications of smart mirrors. This expansion may ultimately benefit long-term health monitoring outside of the clinic, which would be particularly valuable for communities with limited access to healthcare due to social, economic, and/or other barriers. Smart mirrors can be the hub in a digital environment that focuses on full healthcare monitoring, with individuals deploying more sensors to make other general pieces of furniture, such as chairs, tables, and toilets, more intelligent.

## References

[1] Helal S (2005). The gator tech smart house: a programmable pervasive space. Computer.

[2] Enoch JM (2006). History of mirrors dating back 8000 years. Optom. Vis. Sci..

[3] Anwar Hossain, M., Atrey, P. K. & El Saddik, A. Smart mirror for ambient home environment. In *The 3rd IET International Conference on Intelligent Environments* 589–596 (IET Conference Publications, Ulm, 2007).

[4] Pan, J., Appia, V., Villarreal, J., Weaver, L. & Kwon, D.-K. Rear-stitched view panorama: a low-power embedded implementation for smart rear-view mirrors on vehicles. In *Proc. of the IEEE Conference on Computer Vision and Pattern Recognition Workshops* 20–29 (2017).

[5] Nissan Motor Co. Intelligent Rearview Mirror. *Nissan Technological Development Activities* https://www.nissan-global.com/EN/TECHNOLOGY/OVERVIEW/smart_rearview_mirror.html. Accessed 20 March 2018.

[6] Memomi: A. Memory Mirror. http://memorymirror.com/. Accessed 20 March 2018.

[7] Shameer Khader, Badgeley Marcus A., Miotto Riccardo, Glicksberg Benjamin S., Morgan Joseph W., Dudley Joel T. (2016). Translational bioinformatics in the era of real-time biomedical, health care and wellness data streams. Briefings in Bioinformatics.

[8] Insel TR (2017). Digital phenotyping: technology for a new science of behavior. JAMA.

[9] Mango Mirror - Reflect your best life! https://www.mangomirror.com/. Accessed 19 April 2018

[10] Akhlaq M, Sheltami TR, Helgeson B, Shakshuki EM (2012). Designing an integrated driver assistance system using image sensors. J. Intell. Manuf..

[11] Beck M, Crié D (2018). I virtually try it … I want it ! Virtual fitting room: a tool to increase on-line and off-line exploratory behavior, patronage and purchase intentions. J. Retail. Consum. Serv..

[12] Poh MZ, McDuff D, Picard R (2011). A medical mirror for non-contact health monitoring. ACM SIGGRAPH 2011 Emerg. Technol..

[13] Poh MZ, McDuff DJ, Picard RW (2010). Non-contact, automated cardiac pulse measurements using video imaging and blind source separation. Opt. Express.

[14] Besserer, D. et al. Fitmirror: a smart mirror for positive affect in everyday user morning routines. In *Proc. of the Workshop on Multimodal Analyses Enabling Artificial Agents in Human-Machine Interaction* 48–55 (ACM, New York, NY, 2016).

[15] Colantonio S (2015). A smart mirror to promote a healthy lifestyle. Biosyst. Eng..

[16] Andreu Y (2016). Wize Mirror - a smart, multisensory cardio-metabolic risk monitoring system. Comput. Vis. Image Underst..

[17] Picard RW (2009). Future affective technology for autism and emotion communication. Philos. Trans. R. Soc. Lond. B. Biol. Sci..

[18] Zhan Andong, Mohan Srihari, Tarolli Christopher, Schneider Ruth B., Adams Jamie L., Sharma Saloni, Elson Molly J., Spear Kelsey L., Glidden Alistair M., Little Max A., Terzis Andreas, Dorsey E. Ray, Saria Suchi (2018). Using Smartphones and Machine Learning to Quantify Parkinson Disease Severity. JAMA Neurology.

[19] Gomez-Carmona, O. & Casado-Mansilla, D. SmiWork: an interactive smart mirror platform for workplace health promotion. In *2nd International Multidisciplinary Conference on Computer and Energy Science (SpliTech), 2017* 1–6 (IEEE, Split, 2017).

[20] Muse ED, Barrett PM, Steinhubl SR, Topol EJ (2017). Towards a smart medical home. Lancet.

[21] LeCun Y, Bengio Y, Hinton G (2015). Deep learning. Nature.

[22] Kim, J., Lee, J. K. & Lee, K. M. Deeply-recursive convolutional network for image super-resolution. *arXiv [cs.CV]* (2015).

[23] Ledig, C. et al. Photo-realistic single image super-resolution using a generative adversarial network. *arXiv preprint* (2016).

[24] Dahl, R., Norouzi, M. & Shlens, J. Pixel recursive super resolution. *arXiv [cs.CV]* (2017).

[25] Karras, T., Aila, T., Laine, S. & Lehtinen, J. Progressive growing of GANs for improved quality, stability, and variation. *arXiv [cs.NE]* (2017).

[26] Miotto, R., Wang, F., Wang, S., Jiang, X. & Dudley, J. T. Deep learning for healthcare: review, opportunities and challenges. *Brief. Bioinformatics***bbx044**, (2017). 10.1093/bib/bbx044 PMC6455466 28481991

[27] Litjens G (2017). A survey on deep learning in medical image analysis. Med. Image Anal..

[28] Yeung S, Downing NL, Fei-Fei L, Milstein A (2018). Bedside computer vision - moving artificial intelligence from driver assistance to patient safety. N. Engl. J. Med..

[29] Johnson KW (2018). Artificial intelligence in cardiology. J. Am. Coll. Cardiol..

[30] Quer G, Muse ED, Nikzad N, Topol EJ, Steinhubl SR (2017). Augmenting diagnostic vision with AI. Lancet.

[31] Poplin R (2018). Prediction of cardiovascular risk factors from retinal fundus photographs via deep learning. Nat. Biomed. Eng..

[32] Gulshan Varun, Peng Lily, Coram Marc, Stumpe Martin C., Wu Derek, Narayanaswamy Arunachalam, Venugopalan Subhashini, Widner Kasumi, Madams Tom, Cuadros Jorge, Kim Ramasamy, Raman Rajiv, Nelson Philip C., Mega Jessica L., Webster Dale R. (2016). Development and Validation of a Deep Learning Algorithm for Detection of Diabetic Retinopathy in Retinal Fundus Photographs. JAMA.

[33] Esteva Andre, Kuprel Brett, Novoa Roberto A., Ko Justin, Swetter Susan M., Blau Helen M., Thrun Sebastian (2017). Dermatologist-level classification of skin cancer with deep neural networks. Nature.

[34] A skin cancer melanoma detection App. *SkinVision*. https://www.skinvision.com/. Accessed 22 March 2018.

[35] Toshev, A. & Szegedy, C. Deeppose: Human pose estimation via deep neural networks. In *Proc. of the IEEE Conference on Computer Vision and Pattern Recognition* 1653–1660 (2014).

[36] Jain Arjun, Tompson Jonathan, LeCun Yann, Bregler Christoph (2015). MoDeep: A Deep Learning Framework Using Motion Features for Human Pose Estimation. Computer Vision -- ACCV 2014.

[37] Güler, R. A., Neverova, N. & Kokkinos, I. DensePose: dense human pose estimation in the wild. *arXiv [cs.CV]* (2018).

[38] Cvetkoska, B., Marina, N., Bogatinoska, D. C. & Mitreski, Z. Smart mirror E-health assistant—Posture analyze algorithm proposed model for upright posture. In *IEEE EUROCON 2017-17th International Conference on Smart Technologies* 507–512 (IEEE, Ohrid, 2017).

[39] Moon, Y. B. et al. Smart mirror health management services based on iot platform. In *Proc. of the 14th International Conference on Applications of Computer Engineering* 87–89 (2013).

[40] Choi, E., Bahadori, M. T., Schuetz, A., Stewart, W. F. & Sun, J. Doctor AI: Predicting Clinical Events via Recurrent Neural Networks. *arXiv [cs.LG]* (2015). PMC5341604 28286600

[41] Miotto R, Li L, Kidd BA, Dudley JT (2016). Deep Patient: an unsupervised representation to predict the future of patients from the electronic health records. Sci. Rep..

[42] Dunn Jessilyn, Runge Ryan, Snyder Michael (2018). Wearables and the medical revolution. Personalized Medicine.

[43] Hippocrate, A. A. E., Luhanga, E. T., Masashi, T., Watanabe, K. & Yasumoto, K. Smart Gyms Need Smart Mirrors: Design of a Smart Gym Concept Through Contextual Inquiry. In *Proc. of the 2017 ACM International Joint Conference on Pervasive and Ubiquitous Computing and Proceedings of the 2017 ACM International Symposium on Wearable Computers* 658–661 (ACM, Honolulu, 2017).

[44] Nazari Khanamiri, H., Nakatsuka, A. & El-Annan, J. Smartphone Fundus Photography. *J. Vis. Exp*. **125**, 55958 (2017). 10.3791/55958 PMC5609317 28715396

[45] Raju B, Raju NSD (2015). Regarding fundus imaging with a mobile phone: a review of techniques. Indian J. Ophthalmol..

[46] Sankar PL, Parker LS (2016). The Precision Medicine Initiative’s All of Us Research Program: an agenda for research on its ethical, legal, and social issues. Genet. Med..

[47] McConnell MV (2017). Feasibility of obtaining measures of lifestyle from a smartphone app: the MyHeart counts cardiovascular health study. JAMA Cardiol..

[48] Althoff T (2017). Large-scale physical activity data reveal worldwide activity inequality. Nature.

[49] Ten Haken I, Ben Allouch S, van Harten WH (2018). The use of advanced medical technologies at home: a systematic review of the literature. BMC Public Health.

